# Gut microbiota manipulation with prebiotics in patients with non-alcoholic fatty liver disease: a randomized controlled trial protocol

**DOI:** 10.1186/s12876-015-0400-5

**Published:** 2015-12-03

**Authors:** Jennifer E. Lambert, Jill A. Parnell, Bertus Eksteen, Maitreyi Raman, Marc R. Bomhof, Kevin P. Rioux, Karen L. Madsen, Raylene A. Reimer

**Affiliations:** Faculty of Kinesiology, University of Calgary, 2500 University Dr. NW, Calgary, AB T2N 1N4 Canada; Health and Physical Education, Mount Royal University, 4825 Mount Royal Gate SW, Calgary, AB T3E 6K6 Canada; Snyder Institute for Chronic Diseases, Health Research and Innovation Center, University of Calgary, 3280 Hospital Drive NW, Calgary, AB T2N 4N1 Canada; Division of Gastroenterology and Hepatology, Department of Medicine, University of Calgary, 3280 Hospital Drive NW, Calgary, AB T2N 4N1 Canada; Department of Microbiology and Infectious Diseases, University of Calgary, 1863 Health Sciences Centre, 3330 Hospital Drive NW, Calgary, AB T2N 4N1 Canada; Division of Gastroenterology, Centre of Excellence for Gastrointestinal Inflammation and Immunity Research, 7–142 Katz Group–Rexall Centre, University of Alberta, Edmonton, AB T6G 2C2 Canada; Department of Biochemistry & Molecular Biology, Cumming School of Medicine, 3330 Hospital Drive NW, Calgary, AB T2N 4N1 Canada

**Keywords:** Prebiotic fiber, Gut microbiota, Fatty liver, Weight loss, Fibrosis

## Abstract

**Background:**

Evidence for the role of the gut microbiome in the pathogenesis of non-alcoholic fatty liver disease (NAFLD) is emerging. Strategies to manipulate the gut microbiota towards a healthier community structure are actively being investigated. Based on their ability to favorably modulate the gut microbiota, prebiotics may provide an inexpensive yet effective dietary treatment for NAFLD. Additionally, prebiotics have established benefits for glucose control and potentially weight control, both advantageous in managing fatty liver disease. Our objective is to evaluate the effects of prebiotic supplementation, adjunct to those achieved with diet-induced weight loss, on heptic injury and liver fat, the gut microbiota, inflammation, glucose tolerance, and satiety in patients with NAFLD.

**Methods/design:**

In a double blind, placebo controlled, parallel group study, adults (BMI ≥25) with confirmed NAFLD will be randomized to either a 16 g/d prebiotic supplemented group or isocaloric placebo group for 24 weeks (*n* = 30/group). All participants will receive individualized dietary counseling sessions with a registered dietitian to achieve 10 % weight loss. Primary outcome measures include change in hepatic injury (fibrosis and inflammation) and liver fat. Secondary outcomes include change in body composition, appetite and dietary adherence, glycemic and insulinemic responses and inflammatory cytokines. Mechanisms related to prebiotic-induced changes in gut microbiota (shot-gun sequencing) and their metabolic by-products (volatile organic compounds) and *de novo* lipogenesis (using deuterium incorporation) will also be investigated.

**Discussion:**

There are currently no medications or surgical procedures approved for the treatment of NAFLD and weight loss via lifestyle modification remains the cornerstone of current care recommendations. Given that prebiotics target multiple metabolic impairments associated with NAFLD, investigating their ability to modulate the gut microbiota and hepatic health in patients with NAFLD is warranted.

**Trial registration:**

ClinicalTrials.gov (NCT02568605) Registered 30 September 2015

## Background

Non-alcoholic fatty liver disease (NAFLD) is now the most common cause of chronic liver disease worldwide [1]. NAFLD can progress from simple steatosis to non-alcoholic steatohepatitis (NASH) and finally to cirrhosis and its complications (e.g. hepatocellular carcinoma) [1]. Currently the prevalence of NAFLD is 20-30 % of the general population in affluent countries [2–4]. Since NAFLD is a pathological condition closely associated with obesity and insulin resistance [5], it could be expected that rates will continue to rise in conjunction with the growing severity of obesity [6].

### NAFLD Pathogenesis

In 1998, Day and James [7] proposed a ‘two hit’ hypothesis to describe the pathogenesis of NAFLD whereby insulin resistance [8] contributes to steatosis (first hit), which sensitizes the liver to oxidative stress (second hit) leading to inflammation, fibrosis and necrosis. Additional theories, such as the multiple-hit hypothesis, suggest factors including adipokines and mitochondrial dysfunction may also contribute to NAFLD [9]. Unrestrained free fatty acid flux from adipose tissue has been implicated as a primary contributor to steatosis [10, 11], however recent work has indicated that *de novo* lipogenesis is significantly elevated in NAFLD patients and contributes to both plasma and hepatic fatty acid levels [12–14].

The prevalence of NAFLD is estimated to be as high as 75 % in individuals with obesity, 93 % in individuals with morbid obesity, and 47-87 % in people with type 2 diabetes [1]. From a clinical perspective, it is not the simple steatosis that is of concern, but rather the progression to NASH and fibrosis that carries risk for morbidity and mortality [15]. Among individuals with NAFLD, 10 % will progress to NASH, and a third of those patients with early-stage NASH will progress to cirrhosis within 5–10 years, putting tremendous strain on liver transplant registries [16]. In the United States, there was an 8-fold increase in liver transplants attributed to NAFLD between 2001 and 2009 [17]. Aside from hepatic damage, steatosis may accelerate the progression of dyslipidemia, insulin resistance, and atherosclerosis [18], as well as increase the risk for cardiovascular [19] and kidney disease [20]. Given the increased pressure on already over-burdened health care systems, this patient population represents a group in need of effective treatments. As yet, there is a lack of approved treatments for patients with NAFLD, particularly with fibrosis, and therefore investigation of promising targets, including dietary interventions, are critically needed.

### NAFLD Treatment

Currently there are no medications or surgical procedures approved for the treatment of NAFLD. Several agents have been tested, including vitamin E and the thiazolidinediones class of diabetes medications [21–24], though limitations have been identified with both. In the PIVENS (Pioglitazone, Vitamin E, or Placebo for NASH) trial, vitamin E and pioglitazone both improved hepatic steatosis and lobular inflammation but not fibrosis, and pioglitazone was associated with weight gain (4.8 % increase in body weight) [22, 25]. Rosiglitazone improved steatosis but not any other histologic lesions in patients with NASH, and was also associated with weight gain [21].

Weight loss via lifestyle modification remains the foundation for current clinical management of the disease [26, 27]. A reduction in weight of 3-5 % improves biochemical markers and steatosis in NAFLD patients while 10 % weight loss is required for improvement in inflammation and NASH regression [28, 29]. Still, identification of effective and high impact lifestyle interventions to achieve this degree of weight loss is critically needed to provide evidence-informed recommendations.

### Gut microbiota in NAFLD, obesity and associated co-morbidities

Gut microbiota have emerged as an important environmental factor influencing the pathogenesis of NAFLD [9, 30]. Since the liver and intestine are connected anatomically and via the hepatic portal system, the gut microbiota and their metabolic by-products may influence hepatic pathology. The connection between the gut microbiota and NAFLD is incompletely understood, however, and a recent review only identified four animal and five human studies characterising microbial profiles in NAFLD [31]. In mice fed a high fat diet, *Lactobacillus* positively correlated with the severity of hepatic steatosis and the effect was attributed to the impact of *Lactobacillus* on bile acid metabolism [32]. Supplementing a high-fat diet with *Bifidobacterium pseudocatenulatum* CECT 7765 over seven weeks increased *Bifidobacterium* and decreased Enterobacteriaceae; changes associated with a reduction in hepatic steatosis in conjunction with weight loss and improved insulin sensitivity [33]. Finally, inflammasome-deficient mice displayed a dysbiotic microbiome that exacerbated hepatic steatosis and inflammation [34].

In humans, using experimental dietary choline depletion (known to induce hepatic steatosis), higher baseline levels of Gammaproteobacteria correlated with a lower risk for fatty liver. Conversely, Erysipelotrichi correlated with a higher risk, suggesting a person’s microbiome can impact their susceptibility to fatty liver in response to choline deficiency [35]. Quantification of gut microbiota in NAFLD patients has produced conflicting results [36–38] and differences within phyla highlight the need for deeper sequencing. Furthermore, in many studies the effects of NAFLD were not isolated from those of simple obesity. From a treatment perspective, administration of a probiotic combination increased Bacteroidetes and decreased Firmicutes after six months, which correlated with reduced hepatic fat [39]. Further, a meta-analysis evaluating probiotic treatment in NAFLD, found reduced liver enzymes (ALT, AST), TNF-α, total cholesterol and insulin resistance, suggesting that altering gut microbiota may beneficially affect NAFLD [40].

Changes in the gut microbial environment in obesity and type 2 diabetes have been well-documented. These conditions, which are closely tied to NAFLD development, are known to be associated with changes in the vast and diverse communities of microorganisms that live in the human intestinal tract [41–44]. Although not unanimously shown, obesity is associated with a phylum-wide increase in Firmicutes and a reduction in Bacteroidetes [42, 45–47]. This shift is believed to enhance energy harvest, thereby promoting weight gain, hyperglycemia and insulinemia [43, 48–50]. The microbiota also affect the integrity of the gut barrier; in the case of obesity and high fat diets, changes in gut integrity translate to increased intestinal permeability and translocation of endotoxin (lipopolysaccharide from Gram-negative bacteria) [9, 51–53]. Increased leakage of endotoxin into the bloodstream is called metabolic endotoxemia and has been linked to the onset of metabolic diseases [52]. In NAFLD patients, endotoxin levels are elevated [54, 55] and have been associated with small intestinal bacterial overgrowth [9, 56, 57]. The microbiota can also produce endogenous ethanol [9] and ~300 other volatile organic compounds (VOC) [58], some of which may contribute to liver injury. Recently, it has been shown that obese NAFLD patients have an altered microbiome and unique fecal VOC profile compared to healthy controls [38]. While the effect of VOC on liver function is unknown, it is plausible that excess VOC may contribute to a state of elevated inflammation and hepatocyte stress. Given the profound metabolic impact of the gut microbiota to the host, therapeutic manipulation of this community may aid in treatment of NAFLD.

### Prebiotics

Prebiotics are nondigestible food ingredients that are fermented in the gut and modulate the microbiota in a manner beneficial to the host [59–61]. Prebiotic fiber is found naturally in common foods such as asparagus, garlic, leeks and onions [62]. The average intake of prebiotic fiber is estimated to be 1-4 g/d in the Unites States and 3-11 g/d in Europe [63].

There are numerous animal and human studies that point to the potential of prebiotics to treat NAFLD [60, 64–67]. Our lab and others have consistently shown that prebiotic supplementation is associated with lower body weight and/or reduced weight gain in normal [68], genetically obese [69] and high fat, high sucrose-fed [70, 71] rats. We were also the first to report that prebiotic supplementation improves weight loss and reduces energy intake in overweight and obese adults compared to placebo [72], which has since been shown in patients with type 2 diabetes [73]. Increases in satiety hormones observed with prebiotics, including glucagon-like peptide-1 (GLP-1) and peptide YY (PYY) and decreases in the orexigenic hormone ghrelin, likely mediate some of the reduced *ad libitum* energy intake contributing to weight loss [68, 70–72, 74, 75]. These modifications observed with prebiotic supplementation are notable because greater satiety and lower hunger are associated with improved adherence to weight loss interventions [76–79]. Further, the metabolic benefits of prebiotics were evaluated in a recent systematic review of 26 randomized controlled trials [80], where prebiotics were shown to improve satiety, postprandial glucose and insulin in both healthy and obese individuals.

Prebiotics alter gut microbiota in favor of host health [63], including modifying gut barrier integrity and endotoxin translocation. For example, prebiotics increase *Bifidobacterium* which is correlated with lower serum endotoxin levels [81]. In addition, prebiotics stimulate the gut trophic hormone glucagon-like peptide-2, which can modulate endotoxin translocation via effects on epithelial tight junctions [53]. Steatosis itself increases the vulnerability of the liver to injury from endotoxin; while other NAFLD treatment such as vitamin E and thiazolidinediones may reduce steatosis, there is no human evidence that currently suggests vitamin E or the glitazones attenuate endotoxin levels.

The NAFLD Activity Score (NAS) is a histologically-based tool used to grade steatosis, lobular inflammation and ballooning in patients with NAFLD. While weight loss has been shown to reduce NAS, this is largely driven by reductions in steatosis but not ballooning or inflammation, and it does not appear to affect fibrosis [82]. By contrast, we have preliminary histopathological evidence from a pilot trial suggesting that prebiotic fiber improves several hepatic outcomes (i.e. fibrosis) that would augment improvements from weight loss alone (i.e. steatosis) (Unpublished observations; RA Reimer, MR Bomhof). This finding is supported by a recent report that 24 weeks of prebiotic (fructooligosaccharide) plus probiotic (*Bifidobacterium longum*) reduced serum AST, endotoxin and hepatic steatosis in NASH patients [83]. Our recent review [61] highlights convincing evidence from animal and human studies for the potential of prebiotics as a multi-target strategy to treat NAFLD. It is our hypothesis that prebiotic supplementation, as an adjunct to lifestyle-mediated weight loss, will improve hepatic and metabolic pathways beyond those targeted by weight loss alone.

### Specific objectives

The primary objective of this study is to determine the efficacy of a prebiotic supplement in combination with registered dietitian supported weight loss in reducing hepatic injury and liver fat in NAFLD patients over 6 months. Secondary objectives include determining the effect of prebiotic supplementation plus weight loss on appetite, body composition, glucose tolerance, inflammatory cytokines, and potential mechanisms related to gut microbiota, volatile organic compounds, and *de novo* lipogenesis. The research study is novel in that it has been designed to provide comprehensive information on liver health outcomes in NAFLD patients in conjunction with a mechanistic investigation into several of the “multi-hits” thought to promote NAFLD and its progression to NASH.

Given the primary objective of determining the impact of prebiotics on hepatic injury and liver fat in NAFLD patients from baseline to 6 months, patients will undergo a liver MRI, a FibroScan and blood FibroTest. Changes in gut microbiota, VOCs, and LPS will elucidate the effects of prebiotic modulation of the gut microbiota and their metabolic by-products. The impact of prebiotics on obesity-related outcomes will be evaluated through anthropometrics, subjective appetite ratings and satiety hormone responses. Insulin resistance is also closely associated with NAFLD and the effects of prebiotics on glucose regulation will be assessed via responses to an oral glucose challenge. Inflammatory cytokines are another potential “hit” and the effect of prebiotics on plasma inflammatory markers will be quantified. Finally, there is potential for prebiotics to reduce lipogenesis due to its effects on glucose tolerance, food intake, and gut microbiota, and this will be investigated via measurement of *de novo* lipogenesis. This trial is designed to test whether prebiotic supplementation augments the reduction in steatosis attributed to weight loss alone. We hypothesize that the addition of a prebiotic supplement to a registered dietitian-led weight loss intervention, will improve hepatic injury and steatosis to a greater extent than weight loss alone.

## Methods/design

### Ethics, consent and permission

This proposal has been approved (REB14-2464) by the Conjoint Health Research Ethics Board of the University of Calgary (Calgary, AB, Canada). Voluntary, written, informed consent will be obtained from each participant.

### Design

The study is a single-center double blind, placebo controlled, parallel group study. Patients will be randomized to a 16 g/d prebiotic supplemented group or isocaloric placebo group for 24 weeks. All participants will receive individualized dietary counseling sessions with a registered dietitian to achieve 10 % weight loss.

### Inclusion criteria

Adult participants (aged 18–65, BMI ≥25 kg/m^2^) diagnosed with NAFLD on the basis of abnormal liver enzymes (ALT > 1.5x upper limit of normal) and ultrasonography [84] will be recruited from the South Health Campus Fatty Liver Clinic and the surrounding community in Calgary, Alberta, Canada. Individuals with type 2 diabetes treated with diet and exercise alone or metformin will be included, as well as individuals with aspartate aminotransferase and alanine aminotransferase ≤10x upper limit of normal.

### Exclusion criteria

Exclusion criteria include other causes of liver disease such as viral hepatitis and alcoholic liver disease; cirrhosis of the liver (FibroScan >17.5kPa or FibroTest >0.8) or clinical features of cirrhosis; alcohol consumption >20 g/day (2 standard drinks) in women or > 30 g/d (3 drinks) in men; alternate (e.g. TPN) or concomitant etiology for abnormal liver enzymes; history of decompensated liver disease including ascites, encephalopathy or variceal bleeding; concomitant use of any weight loss medication or herbal weight loss products, previous bariatric or other intestinal surgery known to affect food intake or digestive function; presence of active infection, pregnancy or lactation; regular use of a probiotic or prebiotic supplement within 3 months prior to enrollment; antibiotic use within 3 months prior to enrollment; weight loss >3 kg within the preceding 3 months to enrollment; uncontrolled cardiovascular or respiratory disease, active malignancy, or chronic infections; use of agents such as vitamin E, omega-3 fatty acids or medications with evidence for effects on NAFLD (pioglitazone, GLP-1 analogues, dipeptidyl peptidase IV inhibitors, ursodeoxycholic acid); and patients with type 2 diabetes where HbA1c is >9 %.

### Randomization

**Fig. 1:**
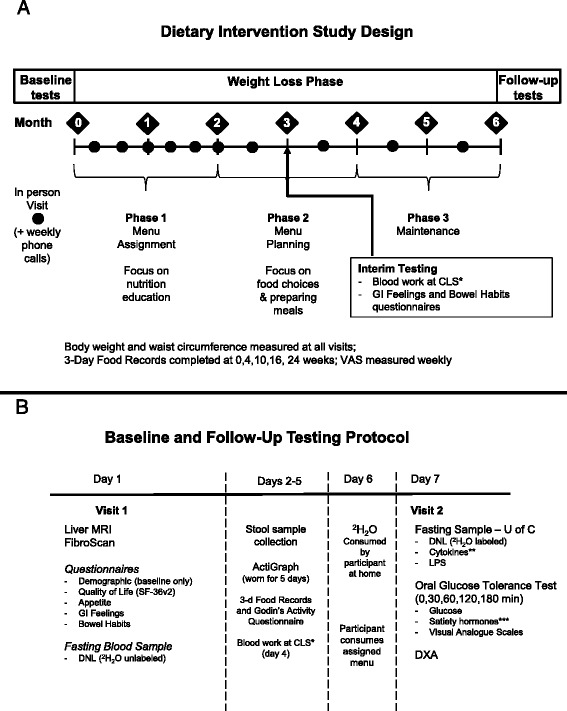
Schematic overviews of the **a** study design and **b** testing day protocols. *Blood work at Calgary Laboratory Services (CLS) includes: glucose, lipids (total, LDL, HDL, TG), HbA1C, liver enzymes (ALT, AST, ALP), FibroTest (gamma-glutamyl transpeptidase, bilirubin, α2-macroglobulin, haptoglobin, and apo-lipoprotein A1), CRP, and liver function biochemistries (creatinine, electrolytes, albumin, bilirubin). **Adipokines/Cytokines include: adiponectin, MCP-1, TNF-α, IL-6 and IL-8. ***Satiety hormones include: active ghrelin, insulin, leptin, total GIP, active GLP-1, and total PYY

Participants will be randomized in equal numbers to two groups, stratified by sex, BMI, and age for allocation into prebiotic or placebo arms. Randomization sequences will be prepared by an independent statistician and entered into a web-based central randomization program. Sequences will not be revealed to investigators or study staff. Randomization will be in blocks of 4 within each sex/age/BMI stratum. To maintain blinding of the trial coordinator and registered dietitian, a research assistant with no involvement in the study will use the central randomization program at the randomization visit. Study personnel will be unaware of treatment allocation prior to the assignment of interventions, using sequentially numbered, opaque, sealed envelopes to maintain allocation concealment. The clinical trial coordinator will enroll participants. Participants, research staff and outcome assessors will be blinded to participants’ assigned group. Participants will receive the prebiotic or placebo supplement in identical foil packets to maintain blinding. An overview of the study design and measurement outcomes is presented in Fig. 1.

Participants will be randomly assigned to a 16 g/day prebiotic dose [Synergy1 (oligofructose-enriched inulin), Beneo-Orafti, Belgium] or an equicaloric dose of placebo [maltodextrin] for 24 weeks. The dose will be ramped up over two weeks and at full dose be consumed as two 8 g packets/day (13.2 kcal/packet) or two 3.3 g packets/day (13.2 kcal/packet) of maltodextrin. Participants will add the packet to 250 ml of water and consume it 30 minutes prior to lunch and dinner. The dose of 16 g/d was selected given previous work showing a reduction in serum AST in NASH patients with prebiotic [85]. Compliance will be assessed via packet counts.

### Dietary weight loss intervention

All participants will be provided diet and lifestyle counseling in one-on-one sessions with a registered dietitian. Participants will be responsible for buying and preparing their own food. The diet will be designed to achieve a weight loss of 0.5-1.0 kg/week with an appropriate energy deficit based on anthropometrics and indirect calorimetry analysis. Participants will meet with the dietitian for 10 in-person visits (1–2 h each). Nutrition education and behavior modification strategies will be used to reduce intake of total energy, added sugars, sodium, and processed food, and to maintain adequate protein intake. Dietary intake during the phases will be assessed by weighed 3-day food records. The dietitian will also contact participants by phone each week to assess adherence, address questions or issues that arise, and monitor body weight. Body weight and waist and hip circumferences will be measured at each visit, and there will be a fasting blood draw at the midpoint of the study (week 12) to monitor blood chemistries (serum aminotransferases and lipids).

During the first phase (visits 1–4), participants will be provided with two 7-day meal plans designed by the dietitian. Meal plans will be personalized for each participants, taking into consideration lifestyle and food preferences based on questionnaires completed at screening. Nutrition education components will include identifying primary food sources of fat, carbohydrate, and protein, reading nutrition labels, and sources of empty calories. In the second phase (visits 5–8), the focus will be on behavior modification and achieving dietary autonomy. This will be achieved through specific counseling which will include planning and preparing meals in the home, portion control, and choosing foods when dining out. Counseling will also be tailored to each individual, based on initial questionnaires related to food craving and behavior. During the final phase (visits 9 and 10), participants will be entirely responsible for planning their own meals and counseling will focus on maintenance of dietary behaviors.

### Primary outcome

#### Hepatic injury and liver fat

The primary outcome is change in hepatic injury and liver fat from baseline to 24 weeks. Improvement in liver injury will be defined by reduction in non-invasive and biochemical markers of fibrosis and inflammation. Fibrosis will be measured at baseline and 24 weeks using transient elastography (FibroScan), which has a high sensitivity for diagnosis and prognostic stratification of varying degrees of liver fibrosis [86, 87] and the Fibrotest which is a composite of serological markers and age and sex, that provides a quantitative estimate of liver damage (age, sex, gamma-glutamyl transpeptidase, bilirubin, α2-macroglobulin, haptoglobin, and apo-lipoprotein A1) [88]. The newer XL probe, specifically designed for obese patients, will be used for FibroScan [86]. Magnetic resonance imaging (MRI) will be performed at baseline and 24 weeks on a 1.5 T scanner (GE Medical Systems, Milwaukee, USA) to assess change in liver fat. MRI can detect fat in microscopic quantities and with chemical shift imaging (CSI) has a sensitivity of 90 % and specificity of 91 % [89]. Although costly, MRI is a front-runner in imaging modalities to quantify hepatic steatosis and represents an acceptable alternative to invasive liver biopsy. While still the gold standard in histological assessment of NAFLD, biopsy is not without risk and is limited by its lack of representation of the liver as a whole [90].

### Secondary outcomes

#### Anthropometrics and Dual Energy X-Ray Absorptiometry

Height will be measured at the beginning of the study to confirm BMI. At each dietitian visit, body weight will be measured using a calibrated balance beam scale along with waist circumference and blood pressure. Body composition will be assessed at baseline and week 24 by DXA (Hologic QDR 4500, Hologic, Inc., Bedford, MA).

### Food record

Food and beverage intake will be assessed using 3-day weighed food records at baseline, 4, 10, 16 and 24 weeks using food scales and standardized forms provided to the participants. Dietary adherence will be defined as prescribed versus measured energy intake. Prior to the start of the study all participants will attend a training session delivered by the dietitian in which they will be instructed on the use of the food scale and how to record their food intake. Participants will then be instructed to weigh and record all food consumed for 2 weekdays and 1 weekend day. This information will be analyzed with FoodWorks software (The Nutrition Company, Long Valley, NJ). Food records will also be used to assess dietary adherence.

### Physical activity and sleep monitoring

Participants will be instructed to maintain their current level of physical activity throughout the study. Physical activity will be monitored at 0, 12, and 24 weeks using a modified Godin’s Leisure Time Exercise Questionnaire that includes duration [91]. Objective monitoring of sleep and physical activity will occur at baseline and 24 weeks. Participants will be provided with an ActiGraph wGT3X-BT (ActiGraph, Pensacola, FL) monitor initialised at 30Hz to wear for five consecutive days. Participants will be instructed to wear the monitor on their waist during waking hours (except when in contact with water) and on their wrist while sleeping, as the monitor has been validated in these positions [92]. At least 3 days of valid wear will be required for the data to be included in the analysis [93]. ActiLife6 software (ActiGraph, Pensacola, FL) will be used to convert data to 1-minute epochs. For physical activity, counts per minute will be classified using predetermined tri-axial vector magnitude cut-points for light, moderate, hard or very hard 0–2690, 2691–6166, 6167–9642, and >9642 counts per min respectively [94]. Sleep will be scored using the Cole Kripke algorithm [95] included in the ActiLife software.

### Subjective and objective assessments of appetite

Subjective ratings of appetite will be recorded by participants at home following a meal, at the same time on the same day, each week using a validated 100 mm VAS [96, 97]. The questions asked based on how the participant felt over the past week are:

1. “How hungry do you feel?” anchored by “I am not hungry at all” and “I have never been more hungry”.
2. “How satisfied do you feel?” anchored by “I am completely empty” and “I cannot eat another bite”.
3. “How strong is your desire to eat” anchored by “I have no desire to eat” and “I have a great desire to eat”.
4. “How full do you feel?” anchored by “Not at all full” and “Totally full”.
5. “How much do you think you could eat?” anchored by “Nothing at all” and “A lot”.
6. Over the course of the past week, my appetite was: “Much lower than usual” and “Much higher than usual”.

### Quality of life and gastrointestinal feelings

Quality of life will be measured at baseline and 24 weeks with the SF-36v2 Health Survey questionnaire [98]. This is a 36 question self-administered questionnaire used to measure functional health and well-being. Bowel habits and gastrointestinal feelings will be assessed at baseline, 12 and 24 weeks using our standard gastrointestinal feelings ratings form (abdominal comfort, bloating, flatulence, rumbling of stomach) and bowel habits questionnaire (number of bowel movements and Bristol Stool Chart).

### Oral glucose tolerance test (OGTT)

Participants will consume a standardized menu the day prior to the OGTT to ensure consistency in macronutrient consumption prior to testing. The menu has been designed by a dietitian and includes 3 meals and 2 snacks. Participants will be instructed to finish dinner and then to not eat after 8 pm. The following morning, a baseline blood sample will be taken via a cannula inserted into the antecubital vein. Subsequently, participants will consume a 75 g oral glucose drink and blood samples will be drawn at 30, 60, 120 and 180 minutes. The OGTT will be conducted at baseline and follow-up (24 weeks). Blood will be centrifuged and plasma frozen at −80 °C for future analyses. Glucose will be quantified using a glucose trinder assay (Stan Bio, Boerne, TX, USA) as per our previous work [72].

### Plasma lipids, liver biochemistry, and inflammatory markers

At baseline, 12 and 24 weeks, a fasting blood sample will be taken for measurement of plasma HbA1c, lipids, CRP, liver enzymes (ALT, ALP, AST), FibroTest, and biochemical markers of liver function (creatinine, electrolytes, albumin, bilirubin). Blood will be drawn at and analysed by Calgary Laboratory Services (Calgary, AB, Canada). Serum adipokines and inflammatory markers (adiponectin, MCP-1, TNF-α, IL-6 and IL-8) will be quantified using Milliplex kits (Millipore, Billerica, MA) from fasting samples obtained at baseline and follow-up (24 weeks) testing.

### Satiety hormones

At each of the five time points during the OGTT, additional blood samples will be collected for measurement of satiety hormones including insulin. Blood will be drawn into a cooled EDTA vacutainer tube containing diprotinin-A (0.034 mg/ml blood; MP Biomedicals, Irvine, CA); Sigma protease inhibitor (1 mg/ml blood; Sigma Aldrich, Oakville, ON, Canada) and Roche Pefabloc (1 mg/ml of blood; Roche, Mississauga, ON, Canada) [72]. Blood will be centrifuged within 30 min and plasma analyzed for ghrelin (active), insulin, leptin, glucose-dependent insulinotropic polypeptide (GIP) (total), GLP-1 (active), and PYY (total) concentrations using a Milliplex MAP Human Gut Hormone Panel Kit (Millipore, St Charles, MO). Surrogate markers of insulin resistance, HOMA and QUICKI, will be quantified as per our previous work [99].

### Mechanistic secondary outcomes

#### Gut Microbiota

Stool will be collected at baseline and follow-up for analysis of gut microbiota. Participants will be instructed on proper methods for stool collection and all materials will be provided in a convenient specimen collection kit. Each subject will collect 2 tablespoons of stool into a pre-labeled sterile container. The container will be sealed, placed in a biohazard bag, and immediately stored in a standard home freezer (−20 °C). The specimen will be transported to our research lab within 4 days of collection in a Styrofoam container on an ice pack and transferred to −80 °C for longer term storage. For analysis of fecal microbiota, total DNA will be extracted from 150 mg of stool using the Qiagen QIAamp™ DNA Stool Mini Kit. Uniform bacterial DNA extraction is ensured by the addition of a 2-minute mechanical bead (0.1 mm zirconia:silica) beating step following addition of buffer ASL to the samples. DNA quantity and purity will be measured using a nanoscale spectrophotometer. To understand the functional and metabolic capabilities of the gut microbiota [100] and how prebiotics impact the metabolic capacity of the entire microbiome, whole genome shot-gun sequencing will be performed to analyze both the microbial composition and potential metabolic functions at the University of Alberta’s Applied Genomic Center. Fecal DNA will be used as input for the Illumina Nextera® XT DNA Sample Preparation Kit to construct indexed paired-end DNA libraries as previously described [101]. A final constructed paired-end indexed library set will be run on a Bioanalyzer 2100 using Agilent High Sensitivity DNA Kit to acquire library average size distribution (Agilent Technologies, Santa Clara). Final libraries will be quantified using a Qubit® 1.0 fluorometer and the Qubit® dsDNA HS assay (Life Technologies, Carlsbad). Obtained quantification and average size distribution of the final Nextera® XT libraries will be used to calculate molarity of the library according to the Nextera® Library Validation and Cluster Density Optimization Technical Note. Libraries are normalized to 2nM and pooled together using equal volume aliquots. The pooled and indexed library set is denatured, diluted, and sequenced on an Illumina MiSeq. Sequencing parameters consist of: paired-end 251 bp dual index sequencing chemistry using MiSeq Reagent Kit-v2 (500 cycle) and FASTQ Only workflow. Produced FASTQ files are then subjected to bioinformatics analysis. The raw FASTQ files are first filtered for adapter sequences and end trimmed of bases with quality less than 15. Read ends are then aligned against a set of mitochondrial databases with SOAPalign, with unaligned reads passed to each successive alignment. Read ends unaligned to mitochondrial databases are aligned to human, bacterial, and viral databases generated from NT, using SOAPalign. SpecI (http://vm-lux.embl.de/~kultima/MOCAT/) will be used to determine species composition and HUMAnN (http://huttenhower.sph.harvard.edu/humann) used for the characterization of microbial pathways in the communities.

### Endotoxin

Serum endotoxin will be measured using a PyroGene recombinant factor C endotoxin detection kit (Lonza, Walkersville, MD) as per our previous work [75].

### Volatile organic compounds

Functional capabilities of microbiota will be assessed in terms of bacterial production of VOC. At the time of analysis, fecal samples will be thawed and a carboxen/polydimethyl-siloxane solid phase micro-extraction (SPME) fiber will be placed into the sample collection vial by injecting it through the septum in the cap. The SPME fiber will be left in the vial headspace for 20 minutes to fully absorb the VOC. The fiber will then be retracted into the needle and transferred to the injector port for gas chromatographic (GC) analysis. Two GC machines connected in serial are used to resolve VOC that are detected by mass spectrometry (MS). Compounds are then identified by reference to the NIST 08 MS library.

### *De Novo* lipogenesis

Energy reduction and weight loss significantly reduces lipogenesis, contributing to reduction in steatosis [102–106]. As part of our mechanistic investigation, fatty acid synthesis will be measured from fasting plasma samples using the deuterium incorporation method as per established methods [12, 107]. This protocol will be performed at a baseline visit prior to the OGTT (Fig. 1). Briefly, deuterium-labeled water (^2^H_2_O; 99.9 atom %; Cambridge Isotope Laboratories, Inc.; Tewksbury, MA) will be provided to participants at a dose of 1 g/kg body water (estimated as 60 % of body weight) to ingest [107]. On day 1, a fasting blood sample will be taken to measure background ^2^H enrichment, and on day 6, participants will ingest a single dose of ^2^H_2_O, as well as be provided with a second dose diluted in 1.5 L of water to consume at regular intervals over the next 24 h [107]. On day 7 at the inpatient visit, a fasting blood sample will be taken for measurement of ^2^H incorporation into triglyceride (TG) and plasma water. Plasma will be ultracentrifugated to isolate total TG-rich lipoproteins, which at fasting will be primarily VLDL arising from the liver [12, 108]. Samples will be stored at −80 °C until further analysis. Folch extraction and thin layer chromatography will be used to isolate the TG portion, which will be saponified and methylated to prepare fatty acid methyl esters (FAME) for GC/P/IRMS analysis [107]. Samples will be analyzed for ^2^H enrichment in major lipogenic fatty acids (14:0, 16:0, 16:1, 18:0, and 18:1). Proportion of newly-synthesized fatty acid will be calculated for each fatty acid using established equations [107].

### Sample size calculation

Our sample size is based on the primary outcomes of hepatic injury and liver fat. For hepatic injury, we used pilot data from our lab to determine that a sample size of *n* = 19 in each group would have 80 % power to detect a difference in liver stiffness of 4.1 kPa with FibroScan assuming a standard deviation of 4.0 kPa and a 0.025 two-sided significance level. For the Fibrotest [109], a sample size of *n* = 10 per group would have 80 % power to detect a difference in Fibrotest score (0.0 to 1.0) of 0.08 assuming a standard deviation of 0.056 and a 0.025 two-sided significance level. For liver fat measured by MRI [110], a sample size of *n* = 25 per group will have 80 % power to detect a difference in hepatic fat of 4.4 % assuming a standard deviation of 5.0 % and a 0.025 two-sided significance level. Based on these calculations, we will enroll *n* = 25 per group. Assuming a dropout rate similar to that in our previous prebiotic trial [72] of approximately 20 %, we will recruit a total of 60 participants (*n* = 30 per group).

### Statistical analyses

Statistical analyses will be performed using SPSS 22.0 software (IBM, Armonk, NY). Values with skewed distribution will be logarithmically transformed prior to analysis. Baseline characteristics will be compared between groups using chi-square for categorical variables and t-tests for continuous variables. Primary analysis will be performed on an intent-to-treat basis, regardless of subject compliance. The primary outcome measures are change from baseline to 24 weeks in hepatic injury (Fibrotest, FibroScan) and liver fat (percent measured by MRI). ANCOVA, with testing for confounding factors (sex) and potential covariates (BMI, age and fasting insulin) will be used to assess the difference between groups at weeks 0 and 24. Should the main effect of diet be significant, Tukey’s multiple comparison test will be used. Secondary outcomes measured at 0 to 24 weeks (e.g. satiety hormones, body fat, glycemia and insulinemia) will be analyzed with ANCOVA as above. For variables measured over time (e.g. body weight, satiety hormones and glucose at 0, 30, 60, 120 and 180 minutes), a mixed model of repeated measures ANCOVA will be applied to examine the effect of treatment, time and their interaction. Our planned secondary analysis will be per protocol with all participants who complete the 24 week intervention and are compliant based on consumption of ≥80 % the product doses. Cases with missing outcome data will be excluded from analysis. Results will be considered statistically significant (2-sided) at *P* ≤ 0.025 (Bonferroni adjustment for 2 primary outcomes).

Shotgun sequencing and GC-MS produce large datasets profiling microbiota composition and function and VOC metabolites, respectively. Binary as well as abundance data will be used in modeling VOC data. OPL-S coefficients determine the major variables contributing to discrimination in the model. VOC of interest will be identified with 90 % probability by referencing to mass spectral library standards of the National Institute of Standards and Technology (NIST08) database. Bacterial communities will be quantitatively assessed by Bray-Curtis dissimilarity matrix and Shannon-Wiener diversity index. Relative abundance data will be transformed [log(X + 1)] prior to generation of the resemblance matrix. To reduce dimension of explanatory variables and identify bacteria that were potentially acting in groups we will conduct hierarchical clustering analysis using Spearman rank correlation with complete linkage and groups identified based on correlations >0.5. Distinct bacteria tax identified from hierarchical clustering analysis will be compared across groups and time-points using formal parametric/non-parametric statistical methods such as the Dirichlet-multinomial distribution test, Mantel test, NP-Manova etc. We will analyze the prebiotic and placebo participants in terms of fecal microbiota, VOC, lipogenesis, clinical data, and host metabolic, nutritional, and inflammatory parameters using differential correlation analysis. Features will be compared using a Spearman’s rank order correlation coefficient and correlations above a defined threshold will be compared between groups. We will then graph the correlations that are statistically different between the groups to identify the pairs of features (i.e. the different VOC metabolites and bacteria between treatments) that distinguish the responses. The network models will be visualized in the popular open-source Cytoscape software.

## Discussion

The prevalence of NAFLD continues to increase worldwide in parallel with obesity and insulin resistance. Despite a multiplicity of factors working in concert to promote the development and progression of the disease, there is sufficient evidence linking NAFLD with dysbiosis of the gut microbiota to warrant an intervention aimed at prebiotic-mediated manipulation of the gut microbiota. Further support for the immediate need for this trial comes from the realization that there are few approved treatment options for NAFLD patients. While the standard of care for patients with NAFLD focuses on weight loss through diet and exercise, the difficulty in achieving and maintaining weight loss is well documented. Given that dietary prebiotic intake is associated with subjective improvements in satiety, there is the added potential for prebiotics to improve adherence to the weight loss intervention. Although approved therapies for NAFLD are still lacking, capitalizing on the gut microbiota–liver axis may provide new therapeutic targets in future.

The planned double blind, randomized clinical trial will determine whether adding prebiotic to a 6-month weight loss intervention will augment improvements in hepatic steatosis and fibrosis attributed to weight loss alone in the placebo group receiving an identical weight loss intervention but without supplementation. The exploration into the mechanisms of action is critically important in advancing treatment options for NAFLD and this study will specifically address *de novo* lipogenesis and metagenomic analysis of gut microbiota and their associated metabolic by-products (i.e. VOCs). Ultimately this clinical trial will advance our understanding of the magnitude to which NAFLD can be treated with a dietary intervention aimed at modulating the gut microbiota.

The proposed study design using daily packets to be consumed with water prior to a meal is feasible as indicated by our previous research [72] and is similar to soluble fiber supplements already on the market. More promising, however, is the versatility of prebiotics. Foods supplemented with prebiotics are already in production because the physical properties and sweet taste allows manufactures to decrease the caloric value while maintaining the palatability and texture of the food [111]. This ease of incorporation is important given the surge in interest in manipulation of the gut microbiota to prevent or treat conditions such as obesity and inflammatory bowel disease. While other options including fecal transplantation and antimicrobial-based interventions are being tested as viable means to manipulate the gut microbiota, targeted dietary interventions such as prebiotics or probiotics are easy to administer and for the most part have good safety and tolerance records [112]. While both prebiotics and probiotics are promising means to manipulate the gut microbiota, unlike prebiotics, probiotics must remain viable during storage and also be capable of surviving the harsh intestinal environment of the host in order to exert their beneficial effects [113]. Additionally, given the well-established health promoting properties of prebiotics and their tolerability, they could be used not only as a treatment but also as a prophylactic.

This trial will assess the ability of a prebiotic to improve overall health in a group at high-risk for a host of metabolic diseases. Improvement in liver health is expected, as are improvements in other predictors of the metabolic syndrome. Ultimately, the research will provide clinical evidence for a potential low-risk treatment option for NAFLD patients. Should the prebiotic intervention prove beneficial, it could have a significant impact on the ability of health professionals to recommend evidence-based targeted dietary interventions for managing NAFLD.

## Abbreviations

ALP: alkaline phosphatase level
ALT: alanine aminotransferase
ANCOVA: analysis of covariance
AST: aspartate aminotransferase
BMI: body mass index
CRP: C-reactive protein
DXA: dual energy x-ray absorptiometry
FAME: fatty acid methyl esters
GGT: gamma-glutamyl transpeptidase
GIP: glucose-dependent insulinotropic polypeptide
GLP-1: glucagon-like peptide-1
GC-MS: gas chromatography-mass spectroscopy
HbA1c: glycosylated hemoglobin
HOMA-IR: homeostasis model assessment of insulin resistance
IL6: interleukin 6
IL-8: interleukin 8
LPS: lipopolysaccharide
MCP1: monocyte chemoattractant protein-1
NAFLD: non-alcoholic fatty liver disease
NAS: non-alcoholic fatty liver disease activity score
NASH: non-alcoholic steatohepatitis
MRI: magnetic resonance imaging
OGTT: oral glucose tolerance test
PYY: peptide tyrosine tyrosine
QUICKI: quantitative insulin sensitivity check index
SCFA: short chain fatty acids
TG: triglycerides
TNFα: tumor necrosis factor alpha
TPN: total parenteral nutrition
VAS: visual analogue scales
VOC: volatile organic compounds
